# Some Negative Effects of Heat Stress in Feedlot Heifers May Be Mitigated via Yeast Probiotic Supplementation

**DOI:** 10.3389/fvets.2019.00515

**Published:** 2020-01-23

**Authors:** Paul R. Broadway, Jeff A. Carroll, Nicole C. Burdick Sanchez, Matt D. Cravey, Jimmie R. Corley

**Affiliations:** ^1^USDA-ARS, Livestock Issues Research Unit, Lubbock, TX, United States; ^2^Phileo Lesaffre Animal Care, Cedar Rapids, IA, United States

**Keywords:** heat, stress, cattle, yeast, probiotic

## Abstract

This study was designed to determine if supplementation of a combination live yeast and yeast cell wall product in feed could mitigate the negative impacts associated with heat stress (HS). Crossbred, phenotypically similar beef heifers (*n* = 32; BW = 385 ± 43 kg) were fed a standard finishing ration without (CON) or with a combination of a live yeast (1.5 g/hd/d) and yeast cell wall product (2.5 g/hd/d; YEAST; Phileo Lesaffre Animal Care, Milwaukee, WI). After 50 d of supplementation, heifers were transported to an environmentally-controlled facility and placed in individual bleeding stalls after indwelling jugular catheters and vaginal temperature (VT) loggers were inserted. Heifers were kept in thermoneutral (TN) conditions for 48 h [temperature-humidity index (THI) ~67; d 1–2] then were subjected to HS for 4 d (peak THI ~80; d 3–6). From d 2–6, hourly blood samples were collected for serum isolation from 1400 to 1800 h and again from 2200 to 0200 h which represented the daily targeted peak and nadir of THIs. A whole blood sample was collected twice daily at 1400 and 2200 h for complete blood counts (CBC). There was no difference in BW (*P* = 0.14) or ADG (*P* = 0.53) between the treatments during HS. Yeast-supplemented heifers exhibited reduced VT during HS compared to CON heifers (*P* < 0.01). There was no difference in water intake during the TN phase (*P* = 0.25); however, YEAST heifers consumed more water/h (*P* < 0.01) and had increased drinking bouts (*P* < 0.01) during HS compared to CON heifers. Respiration rates (RR) did not differ (*P* = 0.21) during TN, but YEAST heifers tended (*P* = 0.09) to have decreased RR during HS compared to CON heifers. There were no differences between treatments when evaluating CBC parameters (*P* ≥ 0.10). There was a tendency (*P* = 0.08) for greater cortisol in the CON than YEAST heifers during HS; however, glucose (*P* = 0.38) and NEFA (*P* = 0.70) concentrations did not differ. In summary, supplementation of live yeast and yeast cell wall products to feedlot heifers may mitigate some of the negative effects associated with HS in feedlot cattle as observed in decreased RR and VT and increased water intake.

## Introduction

Heat stress is primarily the result of elevated air temperature, but can be intensified by high humidity, thermal radiation, and low air movement (1, 2). Heat dissipation mechanisms associated with heat stress in cattle include a combination of radiation, convection, conduction, and evaporation. However, different environmental conditions across the United States influence heat stress occurrence, potential, and severity along with cattle's ability to dissipate the associated heat load (3). Heat stress in animal agriculture results in decreased productivity and can negatively impact animal well-being (1, 4). Specifically, heat stress may reduce weight gain and feed intake, decrease milk production, increase morbidity and mortality, and decrease reproductive performance (4, 5). Some genotypes of cattle are known to have increased heat tolerance capabilities, such as Brahman cattle; however, productivity and growth performance may not be as efficient as in other beef cattle breeds (6, 7). Heat stress also contributes to major economic losses, with one study reporting annual losses of $897 million in beef cattle production in the 48 contiguous states (4).

Beef producers are constantly seeking ways to alleviate some of the negative effects associated with heat stress that result in decreased performance, animal health, and productivity. One of the ways feedlots attempt to combat heat stress is through shade and access/availability to water (3, 7). Some feedlots utilize shade and sprinkler systems to help prevent heat stress, but developing and maintaining the infrastructure can be very expensive. However, in the current production systems of feedlots within the U.S., there are few other options to alleviate heat stress; therefore, cattle feeders are looking for other viable options that may also enhance performance and health.

Yeast products have been and are currently used in beef cattle production for a plethora of reasons encompassing both performance and health benefits (8), and have also been evaluated during heat stress with varied results. In lactating dairy cows during heat stress, supplementation of yeast cultures decreased body temperature but had little effect on other performance or health parameters (9). Another study in dairy cows reported yeast supplementation enhanced performance during heat stress (10). Limited research has evaluated the interactions of yeast product supplementation and heat stress in beef cattle during the finishing phase; however, some research suggests there may be a performance benefit associated with feeding live yeast and yeast cell wall products during heat stress periods (11, 12). A study by Crossland et al. (13) also reported beneficial effects of active dry yeast during heat stress in feedlot steers. Based on this evidence, it was hypothesized that supplementing cattle with yeast would alleviate some of the negative impact effects of heat stress in feedlot cattle. Therefore, the objective of this study was to determine if supplementation of a combination live yeast/yeast cell wall product would mitigate some of the negative effects associated with heat stress in near finished beef cattle.

## Materials and Methods

All experimental procedures were in compliance with the *Guide for the Care and Use of Agricultural Animals in Research and Teaching* and approved by the Institutional Animal Care and Use Committee of the Livestock Issues Research Unit (Protocol: 2015-03-JAC23).

Heifers (*n* = 32; BW = 386 ± 7.1 kg) were acquired from a commercial feedlot in the Texas panhandle and were assigned to 1 of 2 treatments (16 head/treatment), balancing for BW. Heifers were selected based on BW, phenotypic similarity, and lack of medical treatment and were transported to the USDA-ARS Bovine Immunology Research and Development Complex in New Deal, Texas (~40 km). Heifers were randomly assigned to treatments and either fed a standard feedlot ration typical of southern plains feedlot rations consisting of steam-flaked corn, distillers grain, ground hay, and supplemented micronutrients (CON) or the standard feedlot ration supplemented via top dress with a combination of yeast products (YEAST; 1.5 g/hd/d of live yeast and 2.5 g/hd/d of yeast cell wall product; Phileo Lesaffre Animal Care, Cedar Rapids, IA).

After 50 d of supplementation, heifers were weighed and fitted with indwelling vaginal temperature recording devices (14) along with indwelling jugular vein catheters on d 0. Vaginal temperature was recorded at 5-min. intervals for the duration of the study. Animals were placed in individual stanchions (2.28 m in length, 0.76 m in width, 1.67 m in height) in an environmentally-controlled facility with *ad libitum* access to fresh water (on-demand paddle water system) and their respective treatment diets. The individual stalls allowed for normal postural movements and behaviors without hindering standing, lying, or eating behaviors. Heifers were allowed to acclimate to the facility for a 48-h period (d 1–2) in a thermoneutral (TN) climate (THI 67 ± 4). On d 3–6, the THI of the building was gradually increased beginning at 0800 h in a manner to reach peak THI each day at 1600 h (80 ± 3 THI; HS) and the THI was subsequently decreased beginning at 1800 h in a manner to reach the minimum THI of 76 at 2400 h. The THI pattern aimed to keep the heifers in the moderate to severe heat stress category that mimics environmental conditions often seen in summer months when temperatures do not decrease drastically after sunset. The targeted THI patterns designed to mimic environmental conditions during thermoneutral and heat stress phases were partially based on the Livestock Weather Safety Index using temperature and humidity (2, 15). Therefore, for purposes of this study, a THI of 67 was meant to represent no heat stress, and a THI of 80 was meant to represent moderate to severe heat stress. Blood (serum) samples were collected every h from 1400 to 1800 h and again from 2200 to 0200 h on d 2–6. Serum was collected at each time point and was analyzed for cortisol, glucose, and NEFA. A whole blood sample was collected twice daily (1400 and 2200 h) for complete blood cell count (ProCyte DX Hematology Analyzer; IDEXX, Westbrook, ME). Water intake was collected in real-time throughout the duration of the study. Water intake data included the number of drinks taken per h and the quantity consumed per hour (mLs/h). Cattle were fed twice a day (early morning and early afternoon), and feed disappearance was recorded by weighing refusals from each animal prior to the morning feeding. Additionally, at 1600 and 2400 h daily, respiration rate (breaths/min.) was measured and averaged between the same 2 observers at each timepoint throughout the study. On d 7, heifers were weighed, jugular catheters and vaginal temperature recording devices removed, and heifers were returned to outdoor pens prior to returning to the feedyard.

### Serum Analysis

All serum samples were analyzed in duplicate. Serum cortisol concentrations were determined using a commercially available enzyme immunoassay kit according to the manufacturer's directions (Arbor Assays, Ann Arbor, MI, USA) by comparison of unknowns to standard curves generated with known concentrations of cortisol. The minimum detectable cortisol concentration was 45.4 pg/mL, and the intra- and inter-assay coefficients of variation were 7 and 20%, respectively. Data are presented as ng/ml.

Concentrations of NEFAs were determined by modification of the enzymatic HR Series NEFA-HR (2) assay (Wako Diagnostics, Richmond, VA USA) to fit a 96-well-format as previously described (16). Briefly, 200 μL of the prepared Color Reagent A were added to 5 μL of serum or prepared standards in a 96-well-plate. Plates were incubated at 37°C for 5 min and then the absorbance was read using a spectrophotometer at 550 nm. Next, 100 μL of prepared Color Reagent B was added to all wells on the 96-well-plate. Plates were incubated for an additional 5 min and read for a second time using a plate reader at 550 nm. The plate reader used for this assay (BioTek Powerwave 340; BioTek Instruments, Winooski, VT, USA) has an incubating timing feature and therefore ensured that the sample absorbance was read immediately following the 5-min incubation. A final absorbance was obtained by subtracting the first reading, which was multiplied by a factor of 0.67 to account for changes in volume, from the second reading. The final absorbance values were used for all calculations (i.e., standard curve, sample concentrations). Serum concentrations of NEFAs were determined by comparing unknown samples to a standard curve of known NEFA concentrations.

Glucose concentrations were determined by modification of the enzymatic Autokit Glucose (Wako Diagnostics, Richmond, VA) to fit a 96-well-format as previously described (16). Briefly, 300 μL of prepared working solution was added to 2 μL of serum or prepared standards in a 96-well-plate. Plates were incubated at 37°C for 5 min and absorption was recorded at 505 nm. The plate reader used for this assay (BioTek Powerwave 340; BioTek Instruments, Winooski, VT, USA) has an incubating and timing feature and therefore ensured that the sample absorbance was read immediately following the 5-min incubation. Serum concentrations of glucose were determined by comparing unknown samples to a standard curve of known glucose concentrations. The minimum detectable concentration was 3.8 mg/dL and the intra- and inter-assay coefficients of variation were <14.7 and 12.4%, respectively.

### Statistical Analysis

In this completely randomized design, animals were allocated to 1 of 2 treatments. Animal served as the experimental unit. For blood metabolites and biomarkers, Proc Mixed of SAS (v. 9.4, SAS Institute, Cary, SC) was utilized with repeated measures over time within treatment. Fixed effects included treatment, day, time, and their interactions. Data were also analyzed based on differential TN and HS periods. Water intake data was analyzed by quantity consumed/h and number of visits/h. Quantitative water intake data was analyzed using the MIXED procedure of SAS, and categorical frequency water intake data was analyzed using PROC GLIMMIX. Body temperature measurements were recorded at 5-min. intervals but were collapsed into 1-h intervals prior to analysis. Mean differences were separated at *P* ≤ 0.05 using the PDIFF option.

## Results and Discussion

**Figure 1:**
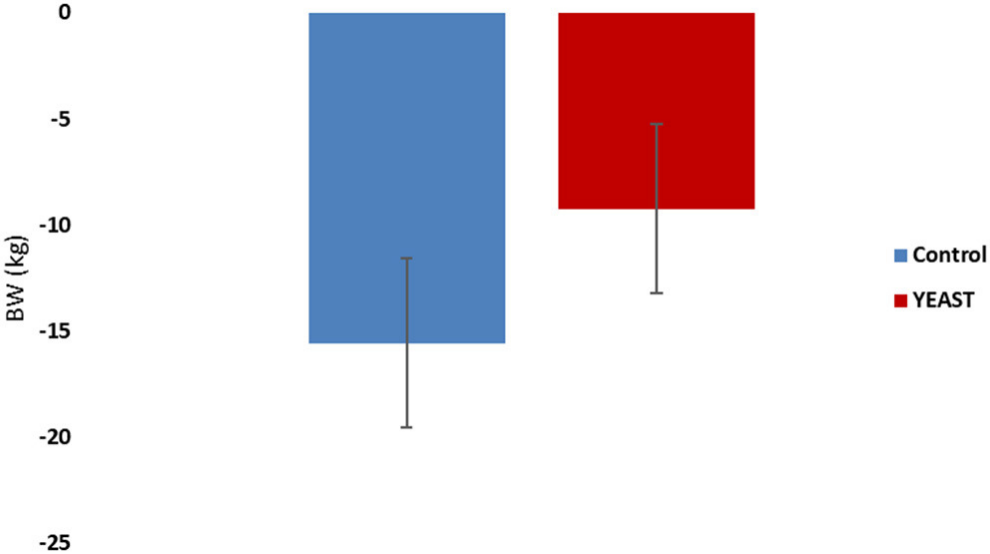
Body weight loss in response to dietary treatment applied prior to and during a heat stress challenge. Heifers were fed one of two dietary treatments: (1) control (CON), fed a standard feedlot diet, or (2) the same standard feedlot diet with 2.5 g·hd^−1^ · d^−1^ live yeast and 1.5 g·hd^−1^ · d^−1^ yeast cell wall product (YEAST) for 33 d prior to the heat stress challenge. Data are presented as LSM ± SEM. There was no difference in body weight change throughout the challenge period (*P* = 0.14).

Body weights of the heifers were collected upon arrival and after the heat stress period. Animals lost weight during the study, which was expected. There was no difference in weight loss between the treatments (−4.19 YEAST vs. −7.06 kg CON; *P* = 0.14; Figure 1). There was also no difference in ADG (*P* = 0.53) between treatments across the 7-d window. Similarly, there was no effect of feed disappearance between the treatments (*P* = 0.15); however, there was an effect of time (*P* < 0.01). Feed disappearance decreased in both treatment groups following the initiation of the heat stress event. The decrease feed intake has been reported previously with elevated THI measurements (17, 18).

**Figure 2:**
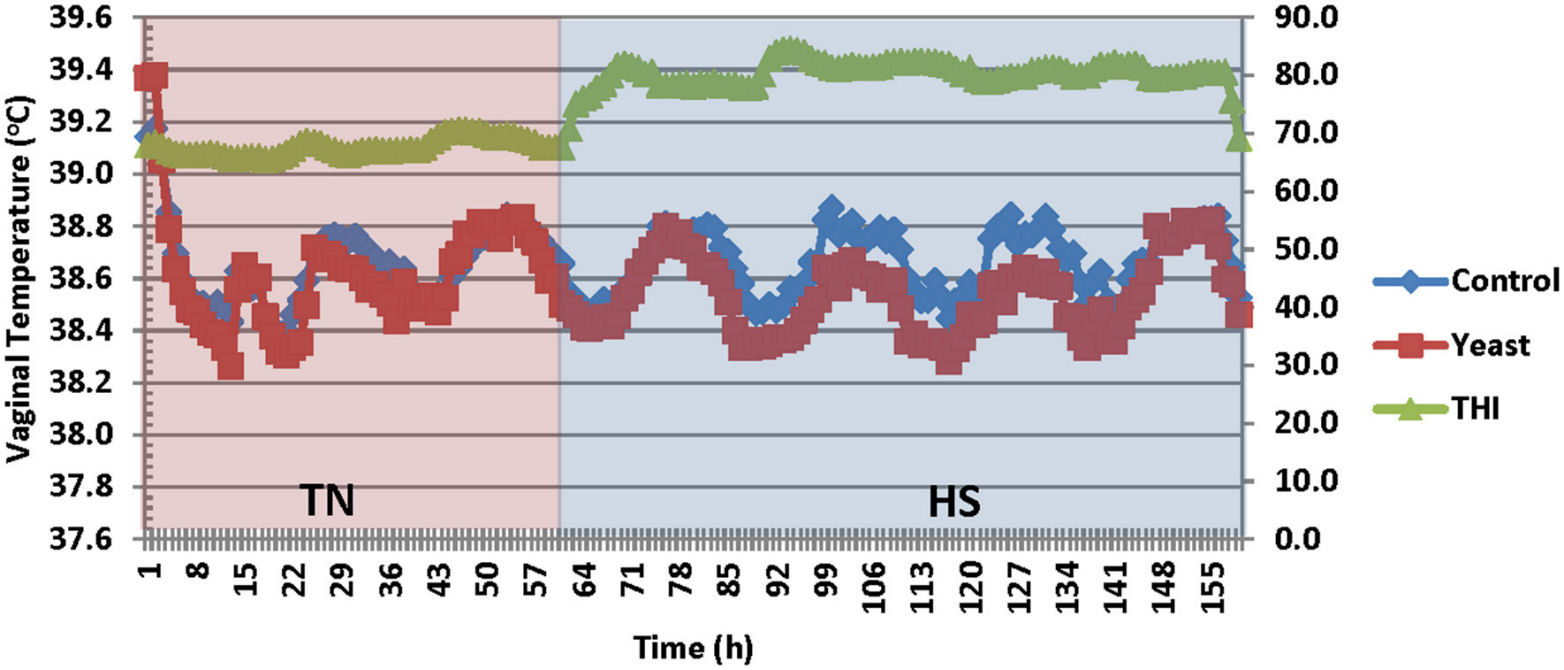
Vaginal temperature in response to dietary treatment applied prior to and during a heat stress challenge. Heifers were fed one of two dietary treatments: (1) control (CON), fed a standard feedlot diet, or (2) the same standard feedlot diet with 1.5 g·hd^−1^ · d^−1^ live yeast and 1.5 g·hd^−1^ · d^−1^ yeast cell wall product (YEAST) for 33 d prior to the heat stress challenge. Data are presented as LSM (SEM: 0.0044 and 0.0047 for CON and YEAST, respectively). THI, Temperature Humidity Index. There was a treatment difference (*P* < 0.01) such that YEAST heifers had decreased vaginal temperature during the heat stress challenge. There was a tendency for a treatment * time interaction (*P* = 0.09).

Vaginal temperatures (VT) were averaged into 1-h intervals prior to analysis from temperature measurements that were collected throughout the study. Overall, the YEAST heifers had reduced VT compared to CON (*P* < 0.01; Figure 2). There was a tendency for a treatment by time interaction (*P* = 0.09) such that VT differences were greater during the heat stress portion of the study in which YEAST heifers maintained a reduced VT compared to CON. Decreased body temperature in yeast supplemented cattle has been observed in previous studies (9, 19, 20). The implications associated with changes in body temperature play a large role in nutrient metabolism. Studies have reported that a 1°C change in body temperature is associated with a 10–13% increase in metabolism (21). Thus, reduced VT, as observed in the yeast-supplemented heifers, may allow for more energy availability for growth, thereby partially mitigating some of the negative impacts of heat stress on feedlot performance, which would correspond to the numerical weight differences between the treatment groups. However, the mechanism by which the YEAST group maintained decreased VT is not fully understood, but may be partially explained by some of the other quantified variables such as water intake.

### Water Consumption and Respiration Rate

**Figure 3:**
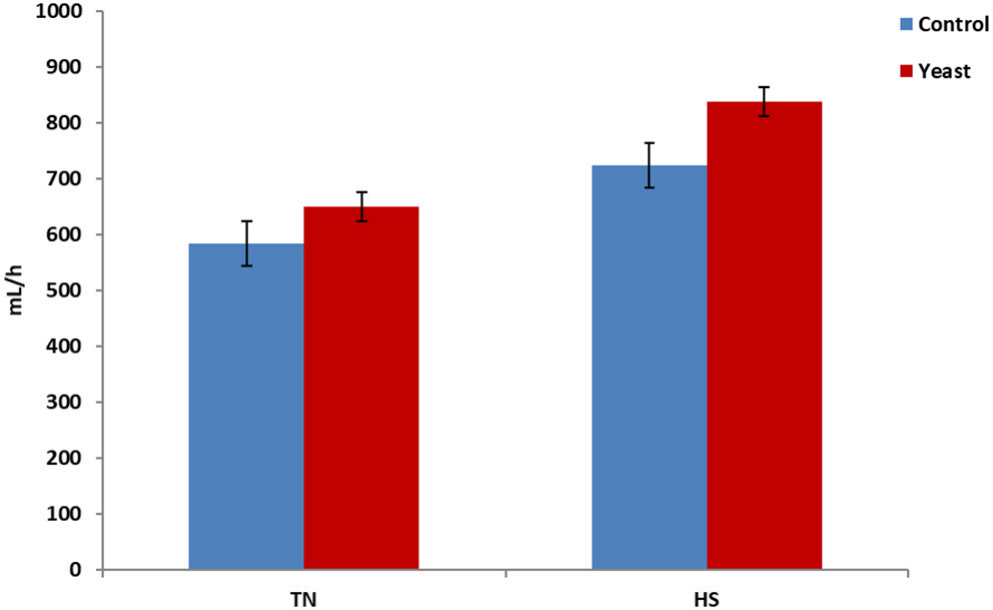
Water consumption per hour in response to dietary treatment applied prior to and during a heat stress challenge. Heifers were fed one of two dietary treatments: (1) control (CON), fed a standard feedlot diet, or (2) the same standard feedlot diet with 2.5 g·hd^−1^ · d^−1^ live yeast and 1.5 g·hd^−1^ · d^−1^ yeast cell wall product (YEAST) for 33 d prior to the heat stress challenge. Data are presented as LSM ± SEM. There was no treatment difference (*P* = 0.25) in water consumption during the thermoneutral phase (TN); however, during the heat stress challenge (HS), YEAST heifers consumed more water than CON heifers (*P* < 0.01).

**Figure 4:**
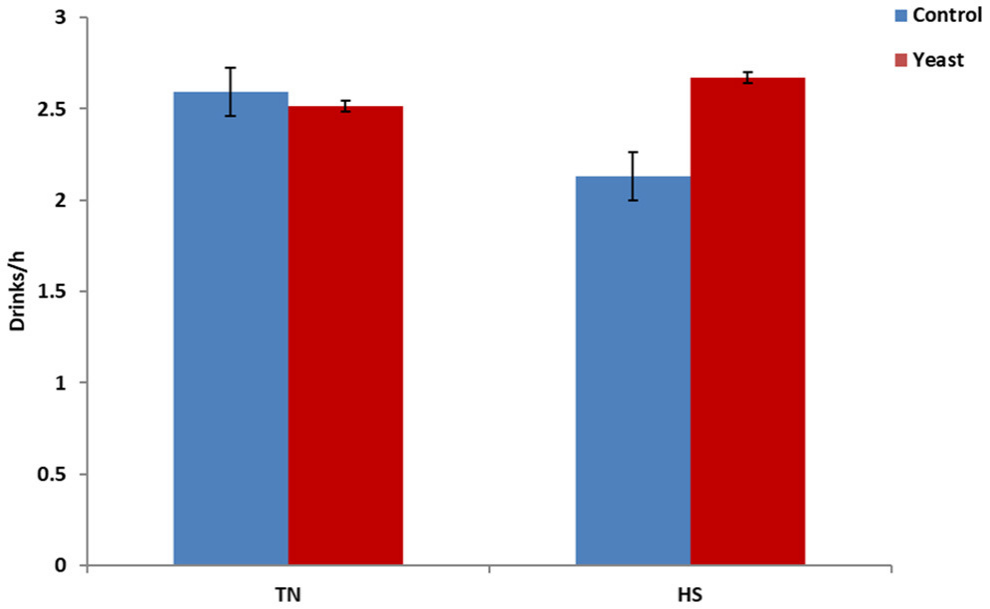
Drinking bouts per hour in response to dietary treatment applied prior to and during a heat stress challenge. Heifers were fed one of two dietary treatments: (1) control (CON), fed a standard feedlot diet, or (2) the same standard feedlot diet with 2.5 g·hd^−1^ · d^−1^ live yeast and 1.5 g·hd^−1^ · d^−1^ yeast cell wall product (YEAST) for 33 d prior to the heat stress challenge. Data are presented as LSM ± SEM. There was no treatment difference (*P* = 0.68) in water drinking bouts during the thermoneutral phase (TN); however, during the heat stress challenge (HS), YEAST heifers consumed water more frequently than CON heifers (*P* < 0.01).

Throughout the entire study, YEAST heifers consumed more water (*P* < 0.01) when compared to CON heifers. When dissected further, there was no difference in mLs/h consumed between the treatments during the TN phase of the study (*P* = 0.25). However, during the HS phase, YEAST heifers consumed more water (*P* < 0.01; Figure 3) than CON heifers. The same pattern occurred when evaluating drinking events or bouts such that YEAST heifers drank more frequently throughout the study than CON heifers (*P* ≤ 0.01). There was no treatment difference when evaluating drinking bouts during the TN phase (*P* = 0.68; Figure 4); however, YEAST heifers drank more frequently during the HS phase of the study than CON heifers (*P* < 0.01). Water consumption has been suggested to be the result of DMI and ambient temperature (22). However, little data has been reported on how yeast products may affect drinking patterns. Increased water consumption during the HS phase may partially explain the observed reduction in VT in the YEAST heifers and may also partially explain the numerically reduced weight loss of the YEAST heifers when compared to CON. Yet, further research is necessary in order to understand how yeast supplementation alters water intake, and the overall implications of increased water intake and frequency of bouts during heat stress.

**Figure 5:**
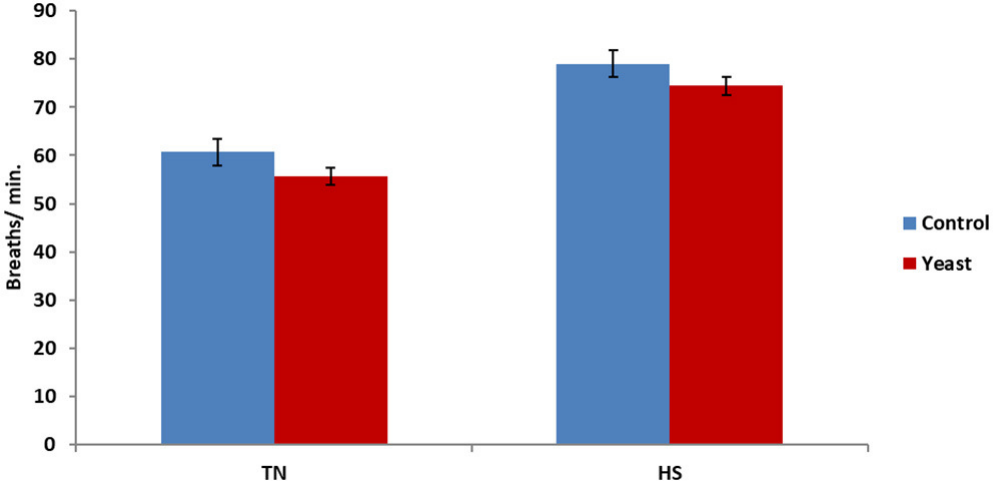
Respiration rate in response to dietary treatment applied prior to and during a heat stress challenge. Heifers were fed one of two dietary treatments: (1) control (CON), fed a standard feedlot diet, or (2) the same standard feedlot diet with 1.5 g·hd^−1^ · d^−1^ live yeast and 2.5 g·hd^−1^ · d^−1^ yeast cell wall product (YEAST) for 33 d prior to the heat stress challenge. Data are presented as LSM ± SEM. There was no treatment difference (*P* = 0.21) in respiration rate during the thermoneutral phase (TN); however, during the heat stress challenge (HS), YEAST heifers tended to have decreased respiration rate when compared to CON heifers (*P* < 0.10).

Overall, there was a tendency (*P* = 0.06) for decreased RR in YEAST heifers. There was no difference in RR during the TN phase (*P* = 0.21; Figure 5); however, there was a tendency (*P* = 0.10) for decreased RR in the YEAST group during the HS phase compared to CON heifers. Panting may partially help alleviate some heat stress via evaporative cooling (18). The tendency for decreased RR in the YEAST group coupled with the aforementioned VT differences between treatments suggests and possibly reaffirms that the YEAST group was not experiencing as much distress during the heat stress. These data differ from a heat stress study utilizing dairy cattle which reported no difference in respiration or sweating rates when feeding a yeast culture (9). While increased panting may theoretically lead to a reduced body temperature, that was not the case in this study, suggesting that some other physiologic systems were involved in thermoregulation due to yeast supplementation.

### Hematology

There were variable effects on hematology variables between treatments. No differences were observed in red blood cells, hemoglobin, platelets, total white blood cells, or basophils (P > 0.05). Heifers in the CON group had decreased hematocrit (*P* = 0.03; Table 1) compared to YEAST heifers. Hematocrit is an indicator of red blood cell volume and oxygen transport availability. This difference may be associated with hydration status and may be partially associated with the increased water intake observed in the YEAST treatment, but further research is necessary in order to confirm this link in cattle. Increased hematocrit is generally associated with dehydration (23); however, water intake and other variables do not support that conclusion in this particular study. Neutrophil counts were reduced in the YEAST group throughout the 6-d sampling period compared to CON heifers (Table 1). This is similar to findings in other studies utilizing similar yeast supplements, where live yeast and/or yeast cell wall products reduced neutrophil counts compared with non-supplemented controls (24). Studies have indicated that components of yeast, such as the beta-glucans, can improve neutrophil function (25, 26). Thus, the reduction in neutrophil concentrations may reflect a reduced requirement for neutrophils in circulation due to enhancement of neutrophil functionality. However, specific assays that assess neutrophil functionality would be required to confirm this theory. Additionally, it appears the reduction in neutrophils may reflect a general effect of the yeast products supplemented in this study on neutrophils, and not necessarily a response to the heat stress challenge. Additionally, heifers in the YEAST group had increased lymphocytes (*P* < 0.01) and eosinophils (*P* < 0.01; data not shown) compared to CON heifers (Table 1). Similar to neutrophils, this may reflect a general response to the yeast supplement and not necessarily a response to the heat stress challenge. Increased lymphocytes and eosinophils in circulation may reflect an increase in these cells in preparation for an ensuing challenge, thus reflecting the effects of yeast as an immunostimulator. This is supported by data in dairy cows that found increased numbers of T and B lymphocytes in cows supplemented with yeast during early lactation (27). Therefore, it appears that while there were few differences in hematology parameters due to treatment, the differences observed follow previous observations in yeast-supplemented animals.

**Table 1:** Blood hematology for circulating blood cell variables in response to dietary treatment applied prior to and during a heat stress challenge.

	**Treatment**			***P*****-value**		
**Variable**	**CON**	**YEAST**	**SEM**	**Treatment**	**Time**	**Treatment** *Time
Hematocrit (%)	34.22	35.02	0.82	0.03	< 0.0001	0.97
Hemoglobin (g/dL)	11.28	11.37	0.23	0.35	< 0.0001	0.99
Neutrophils(K/μL)	4.27	3.81	0.34	0.00	0.01	0.99
Lymphocytes (K/μL)	3.88	4.16	0.23	0.01	0.88	1.00

*Heifers were fed one of two dietary treatments: (1) control (CON), fed a standard feedlot diet, or (2) the same standard feedlot diet with 1.5 g·hd^−1^·d^−1^ live yeast and 2.5 g·hd^−1^·d^−1^ yeast cell wall product (YEAST) for 33 d prior to the heat stress challenge. Data are presented as LSM ± SEM pooled as an average over time*.

### Serum Analysis

**Figure 6:**
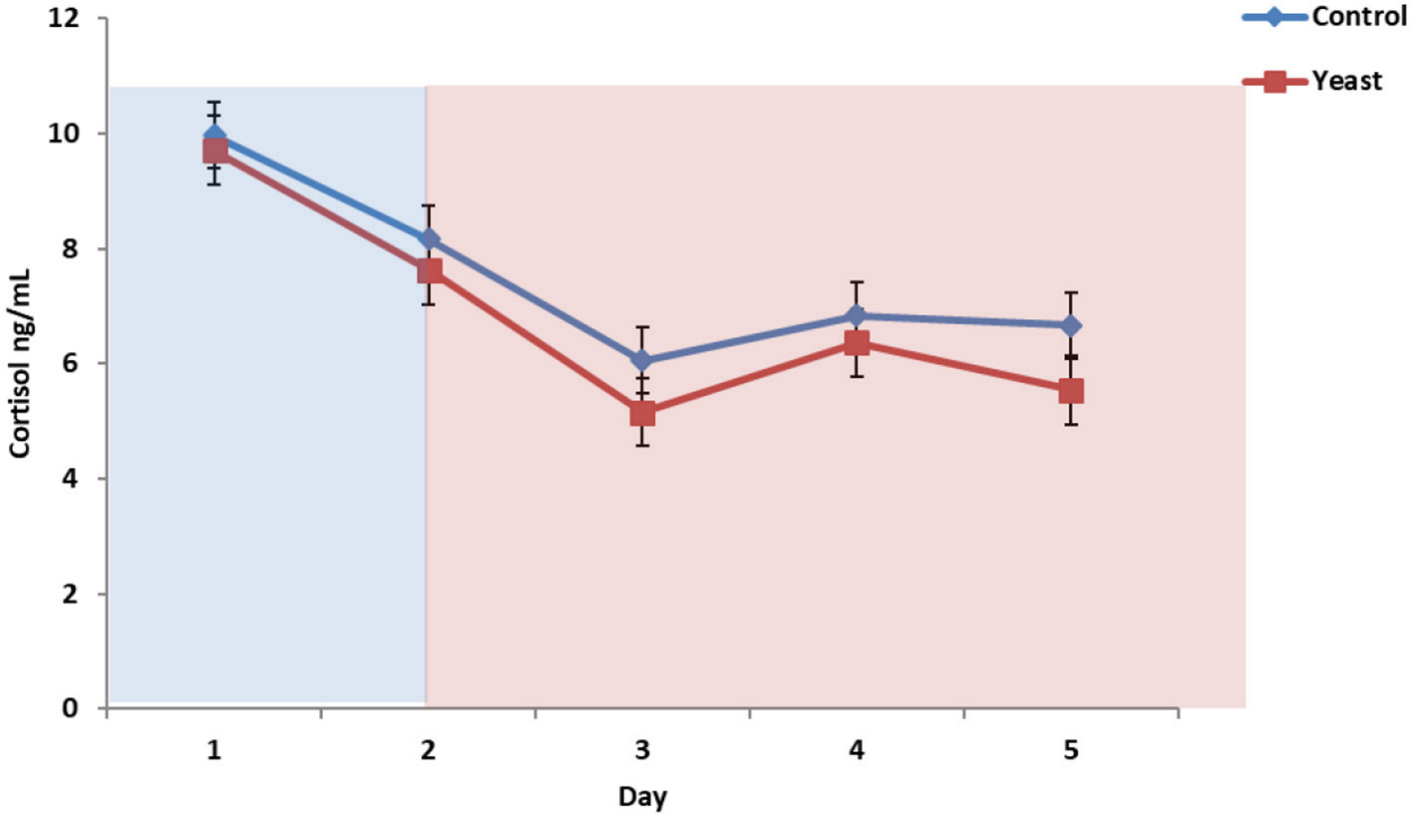
Circulating cortisol concentrations in response to dietary treatment applied prior to and during a heat stress challenge. Heifers were fed one of two dietary treatments: (1) control (CON), fed a standard feedlot diet, or (2) the same standard feedlot diet with 1.5 g·hd^−1^ · d^−1^ live yeast and 2.5 g·hd^−1^ · d^−1^ yeast cell wall product (YEAST) for 33 d prior to the heat stress challenge. Data are presented as LSM ± SEM. Blue shading represents the thermoneutral (TN) period, and red shading represents the heat stress (HS) period. There was a tendency for a difference in circulating cortisol throughout the challenge period (*P* = 0.08).

Overall, CON cattle tended to have more circulating cortisol (*P* = 0.08), and there was no treatment by time interaction (*P* = 0.62; Figure 6). Cortisol remained at baseline concentrations throughout the study (<10 ng/ml). These cortisol concentrations suggest that the heifers were acclimated to the bleeding stalls and were not experiencing stress due to sample collection, feeding, and/or other processes occurring in the facility. Another heat stress study reported elevated cortisol from 20 min to 2 h following initiation of heat stress; however, the authors suggested that chronic exposure may result in suppressed or decreased cortisol (28). Since the bleeding timeline was representative of peak and valley THI, sample collection frequency may not have been conducive for elucidating the cortisol response to heat stress.

**Figure 7:**
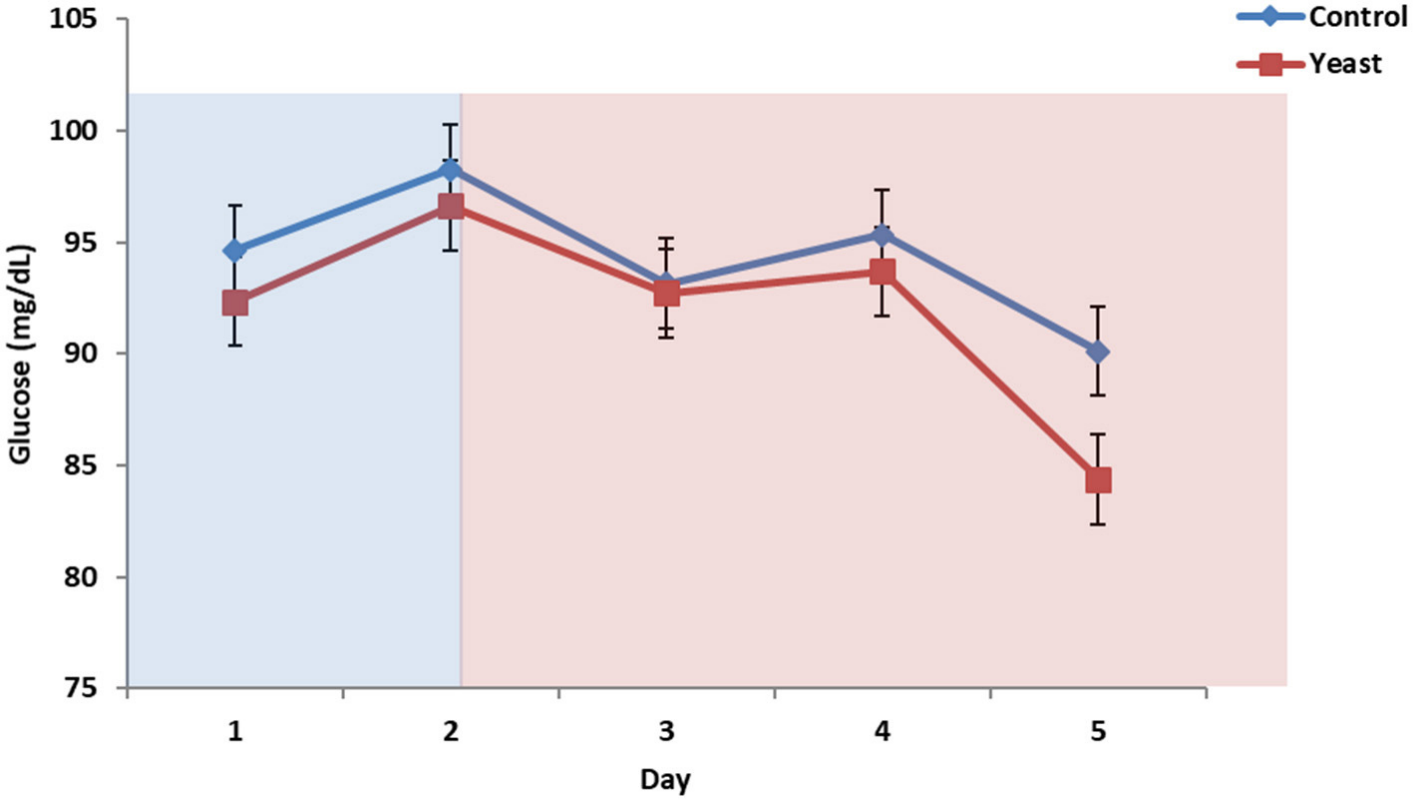
Circulating glucose concentrations in response to dietary treatment applied prior to and during a heat stress challenge. Heifers were fed one of two dietary treatments: (1) control (CON), fed a standard feedlot diet, or (2) the same standard feedlot diet with 1.5 g·hd^−1^ · d^−1^ live yeast and 2.5 g·hd^−1^ · d^−1^ yeast cell wall product (YEAST) for 33 d prior to the heat stress challenge. Data are presented as LSM ± SEM. Blue shading represents the thermoneutral (TN) period, and red shading represents the heat stress (HS) period. There was no difference in change in circulating glucose throughout the challenge period (*P* = 0.95).

**Figure 8:**
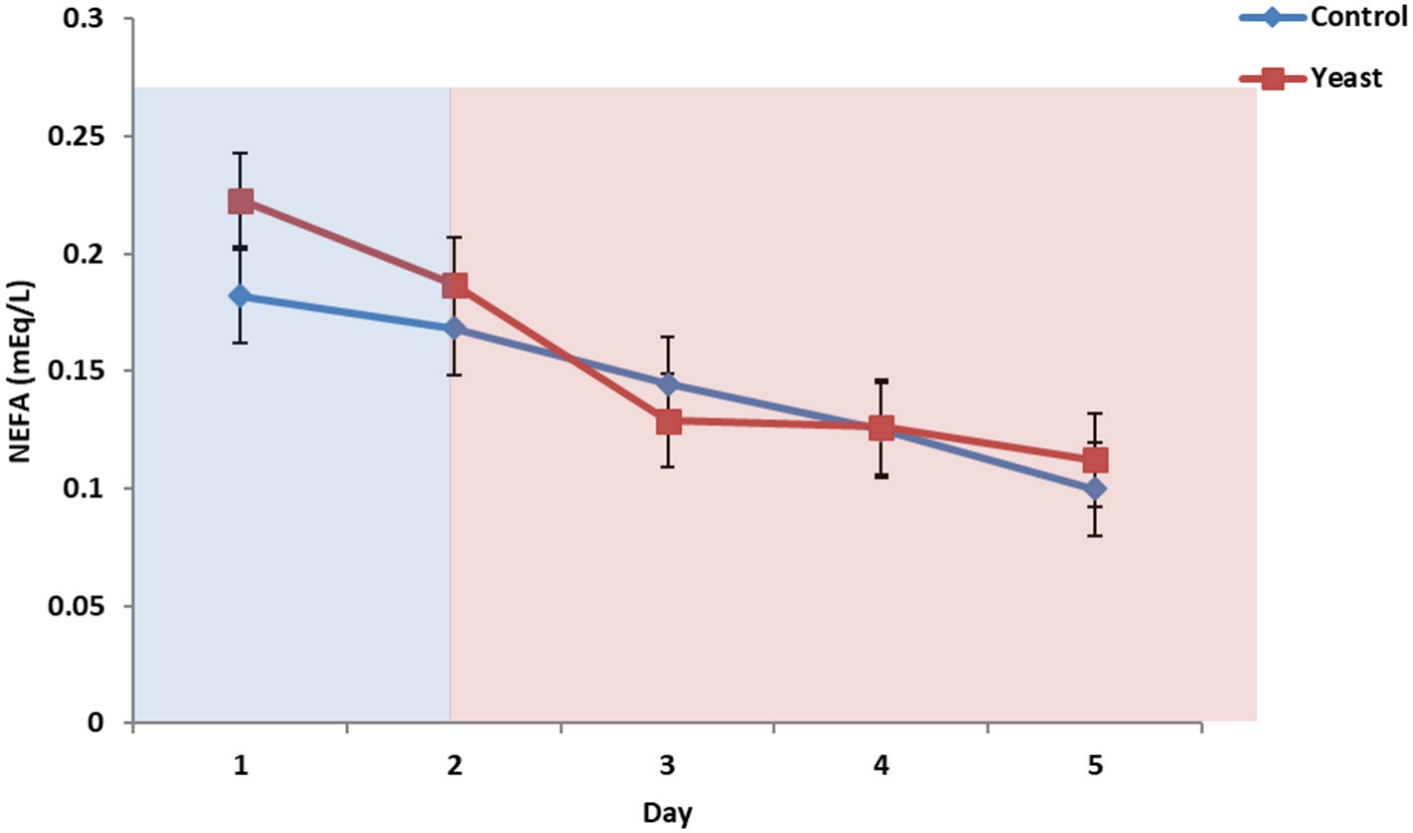
Circulating non-esterified fatty acids (NEFA) concentrations in response to dietary treatment applied prior to and during a heat stress challenge. Heifers were fed one of two dietary treatments: (1) control (CON), fed a standard feedlot diet, or (2) the same standard feedlot diet with 1.5 g·hd^−1^ · d^−1^ live yeast and 2.5 g·hd^−1^ · d^−1^ yeast cell wall product (YEAST) for 33 d prior to the heat stress challenge. Data are presented as LSM ± SEM. Blue shading represents the thermoneutral (TN) period, and red shading represents the heat stress (HS) period. There was a treatment * time interaction for circulating NEFA (*P* < 0.01) such that prior to the challenge, circulating NEFAs were greater in the YEAST heifers; however, there were no differences during the heat stress phase of the study.

No differences were observed in circulating glucose concentrations between treatments (*P* = 0.38; Figure 7). Circulating glucose decreased during heat stress as reported in a study comparing heat stressed cattle to pair-fed growing cattle; however, this observed phenomenon is different depending on species (29). Overall, there were no treatment differences in circulating NEFA concentrations (*P* = 0.70; Figure 8) for the duration of the study. Unpublished data from our laboratory obtained from animals supplemented with yeast products observed decreased serum concentrations of NEFAs during a live pathogen challenge. Another study utilizing heat stressed cattle observed no changes in NEFA concentrations due to heat stress (11, 29, 30). Similar NEFA responses have been observed in pigs (31). Thus, there are varying effects within and across species with regard to circulating NEFA concentrations during heat stress which suggests NEFAs may not be the best marker to measure the catabolic effects of heat stress in cattle.

## Conclusion

Heat stress is a costly problem for the beef cattle industry, and little can be done to alleviate the negative effects associated with it beyond preventative measures. In this study, heifers supplemented with a combined live yeast and yeast cell wall product had decreased VT and respiration rate and increased water intake in comparison to non-supplemented control heifers. Yeast supplemented heifers also lost slightly less weight during heat stress. However, there were minimal effects on other variables. In summary, these data suggest that some of the negative effects of heat stress may be mitigated by yeast and yeast cell wall supplementation prior to a heat stress event but further research is needed to elucidate the complex interactions of yeast supplementation and heat stress, juxtaposed with the physiological changes occurring simultaneously.

## Data Availability Statement

The datasets generated for this study are available on request to the corresponding author.

## Ethics Statement

This animal study was reviewed and approved by this study was carried out in accordance with the Guide for the Care and Use of Agricultural Animals in Research and Teaching and approved by the Institutional Animal Care and Use Committee of the Livestock Issues Research Unit (Protocol: 2015-03-JAC23).

## Author Contributions

PB, JAC, NB, and JRC initiated the initial study design. PB, JAC, NB, and MC performed the study and collected data. Data was analyzed and the manuscript was written by PB. All authors reviewed manuscript drafts, provided constructive comments, and approved the manuscript for submission.

### Conflict of Interest

MC and JRC were employed by Phileo Lesaffre Animal Care. The remaining authors declare that the research was conducted in the absence of any commercial or financial relationships that could be construed as a potential conflict of interest.
